# Characterization of a Chikungunya virus strain isolated from banked patients’ sera

**DOI:** 10.1186/s12985-016-0606-3

**Published:** 2016-09-02

**Authors:** Pattra Chalaem, Sarunyou Chusri, Stefan Fernandez, Wilaiwan Chotigeat, Juan Anguita, Utpal Pal, Kamoltip Promnares

**Affiliations:** 1 Department of Molecular Biotechnology and Bioinformatics, Faculty of Science, Prince of Songkla University, Hatyai, Songkhla 90112 Thailand; 2 Division of Infectious Disease, Department of Internal Medicine, Faculty of Medicine, Prince of Songkla University, Hatyai, Songkhla 90112 Thailand; 3 Department of Virology, Armed Forces Research Institute of Medical Sciences (AFRIMS), Bangkok, Thailand; 4 CIC bioGUNE, 48160 Derio, Bizkaia Spain; 5 Ikerbasque, Basque Foundation for Science, 48011 Bilbao, Bizkaia Spain; 6 Department of Veterinary Medicine and Virginia-Maryland Regional College of Veterinary Medicine, University of Maryland, College Park, MD 20742 USA

**Keywords:** Chikungunya virus, 293T cells, CHIK/SBY8/10 isolate, CHIKV 181/clone 25, Gene expression, Cytokines

## Abstract

**Background:**

Chikungunya virus (CHIKV) is a prevalent mosquito-borne pathogen that is emerging in many parts of the globe causing significant human morbidity. Here, we report the isolation and characterization of an infectious CHIKV from banked serum specimens of suspected patients from the 2009 epidemic in Thailand.

**Methods:**

Standard plaque assay was used for CHIKV isolation from the banked serum specimens. Isolated CHIKV was identified base on E1 structural gene sequence. Growth kinetic, infectivity, cell viability and cytokine gene expression throughout CHIKV infection in a permissive cell line, 293T cells, was performed using several approaches, including standard plaque assay, immunofluorescence assay, classical MTT assay, and quantitative real-time PCR. Two tailed Student’s *t* test was used for evaluation statistically significance between the mean values of the groups.

**Results:**

Based on the E1 structural gene sequence and phylogenetic analysis, we identified the virus as the CHIK/SBY8/10 isolate from Indonesia. Assessment of the growth kinetics, cytopathic effects as well as its ability to induce cellular immune responses suggested that the currently isolated CHIK/SBY8/10 virus is relatively more virulent than a known CHIKV vaccine strain, which also induces more dramatic proinflammatory responses.

**Conclusions:**

Our studies further add to the infectivity of a less-studied yet infectious CHIKV isolate as well as underscored the importance and utility of 293T cells as an excellent cell culture model for studying viral growth, CHIKV-induced inflammatory cellular responses and cell death. Together, these studies provide novel information on the CHIKV biology, infectivity and virus-cell interaction, which would help develop novel interventions against the infection.

## Background

First discovered in Tanzania in African continent in 1952 and later identified in Thailand in 1958, Chikungunya virus (CHIKV) fever is a re-emerging public health problem with significant human morbidity, accounting for approximately 1.4 million suspected cases worldwide [1]. Although the infection is most prevalent in Africa and Asia, travellers from other continents can import the infection locally, including in non-endemic areas; for example, as of 2015, there were over 1,379,788 suspected cases of CHIKV in North and South America including at least 191 deaths [2]. CHIKV infection is caused by the single-stranded, positive-strand RNA virus that is transmitted by *Aedes* mosquitoes [3–6]. While *A. aegypti* was long been considered as the only mosquito vector for CHIKV, investigation in the 2005–2006 epidemic on Reunion island that involved 266,000 human cases suggested that a single mutation, an alanine-to-valine substitution in the E1 envelope glycoprotein at the position 226 enabled virus acquisition by a new mosquito vector, *A. albopictus,* commonly known as the Asian tiger mosquito, which was implicated in that epidemic [7]. Due to already established widespread distribution as well as increasing global expansion of *A. albopictus*, the potential for CHIKV to extend its range into the Western hemisphere is a real possibility [8, 9]. Although CHIKV and dengue fever manifest similar clinical symptoms and are transmitted by the same mosquito vectors reflecting similar epidemiology and geographical distribution [10], CHIKV is relatively less studied and effective diagnostics and vaccines are currently unavailable. Clinical manifestations following CHIKV infection are also similar to those developed upon Zika infection. A mild to severe joint pain is the hallmark of CHIKV infection, and CHIKV is considered one of the most prominent arthritogenic alphavirus. Although intense Th2 cytokine responses are typically associated with the infection [11], studies reported significant correlations between cytokines, anti-CHIKV antibodies and clinical features. The control of the virus during infection is achieved through acquired immune responses; however, the persistence of the virus in joint tissue is associated with its pro-arthritogenic potential, likely inducing the production of proinflammatory factors [12]. Despite these studies, the mechanisms influencing immunopathogenesis of CHIKV infection remains unclear [13, 14].

Approximately 11.8 kB RNA genome of CHIKV encodes four nonstructural proteins (nsP1–4) and a single structural polyprotein, which after proteolytic processing, generates three main structural proteins: the capsid and the envelope glycoproteins E2 and E1, which exists in the virion as a E1/E2 heterodimer [15, 16]. While E2 is responsible for viral attachment to target host cell, E1 is a class II viral fusion protein, which after dissociation from E2, trimerizes and mediates fusion of viral and host cellular membranes [17]. Sequence of Envelope E1 is often used for phylogenetic analysis of CHIKV and is classified into 3 genetic lineages: Asian, West African, and East/Central/South African (ECSA) genotypes [9, 18, 19]. The major outbreak of chikungunya fever in Thailand in 2008–2009, which affected at least 50,000 people was caused by the ECSA lineage virus [20]. Notably, since 2009, despite a large outbreak and more recent human cases, studies addressing characterization of CHIKV clinical isolates in Thailand are still very limited [21]. Here, we report the initial characterization of a CHIKV clinical isolate purified from banked serum specimens of suspected patients from the 2009 epidemic in Thailand. We sequenced the E1 structural gene and characterized the isolated virus in a permissive cell line, 293T cells. We examined the viral growth kinetics and studied the cytopathic effects as well as assessed the ability of the CHIKV isolate to induce cellular cytokine responses. Together, these fundamental studies increase our knowledge of CHIKV biology, virus-cell interaction and will help develop novel interventions against the infection.

## Methods

### Cell cultures

The human embryonic kidney epithelial cell line 293T (Invitrogen, Grand Island, NY, USA) was kindly provided by Dr. Sansanee Noisakran, National Center for Genetic Engineering and Biotechnology, Thailand. 293T cells were cultured at 37 °C, 5 % CO_2_ in Dulbecco’s Modified Eagle Medium (DMEM; Gibco, Invitrogen, USA) supplemented with 10 % heat-inactivated fetal bovine serum (FBS; Gibco, Invitrogen, USA), 37 μg ml^−1^ of penicillin, 60 μg ml^−1^ of streptomycin, 25 mM HEPES, 2 mM L-glutamine, and 0.37 % NaH_2_CO_3_. African green monkey kidney Vero cell (American Type Culture Collection; ATCC, Manassas, VA) and *Aedes albopictus* C6/36 cell [22], were kindly provided by Ms. Tanapan Pruksamas, National Center for Genetic Engineering and Biotechnology, Thailand. Vero cells were cultured at 37 °C, 5 % CO_2_ in Minimum Essential Medium (MEM; Gibco, Invitrogen, USA) supplemented with 10 % heat-inactivated FBS, and 37 μg ml^−1^ of penicillin, 60 μg ml^−1^ of streptomycin. C6/36 cells were cultured at 28 °C in Leibovitz’s L-15 medium (Gibco, Invitrogen, USA) supplemented with 10 % heat-inactivated FBS and 10 % tryptose-phosphate.

### Virus isolation and propagation

Chikungunya viruses (CHIKVs) were isolated from five patient sera collected from Songkhla Province, Thailand from the 2009 outbreak using standard virology procedures [7]. These sera were tested negative for the presence of dengue virus (DENV) and yellow fever virus, as assessed by routine PCR analysis. *A. albopictus* C6/36 cells were inoculated with 200 μl of patient sera and incubated at 28 °C. Cytopathic effects (CPE) were observed daily until appearance of 70–80 % CPE. Supernatants were harvested and serially diluted from 10^−1^ to 10^−4^ in Leibovitz’s L-15 medium (Gibco, Invitrogen, USA) supplemented with 2 % heat-inactivated FBS and 10 % tryptose-phosphate. Each diluted serum was added to monolayer of *A. albopictus* C6/36 cells and gently agitated at room temperature for 2 h. Supernatants were discarded and the Leibovitz’s L-15 medium containing 3 % agarose (Invitrogen, USA) was overlaid. The cells were incubated at 28 °C and CPE were observed daily until appearance of single plaques. Single plaques were picked and resuspended in MEM (Gibco, Invitrogen, USA) supplemented with 2 % heat-inactivated FBS. The resuspended single plaques were inoculated in Vero cells and gently agitated at room temperature for 2 h before incubation at 37 °C, 5 % CO_2_ for 7 days. CPE was observed daily for 7 days. Infected Vero cell suspension at day 7 was aliquoted for confirmation of CHIKV using polymerase chain reaction (PCR). The isolated CHIKV were propagated in Vero cells as described above until the titer reached about 4x107 pfu/ml and were harvested by supplementing the infected Vero cell suspension with 20 % heat-inactivated FBS before filtration through Millex-GP filter (EMD Millipore, USA). The virus was kept at −80 °C until performing subsequent experiments.

### Standard plaque assay

CHIKV 181/clone 25 (herein abbreviated as V-181) was kindly provided by Dr. Butsaya Thaisomboonsuk, the Armed Forces Research Institute of Medical Sciences (AFRIMS), Thailand. V-181 and the isolated CHIKV were serially diluted in MEM (Gibco, Invitrogen, USA) supplemented with 2 % heat-inactivated FBS. The diluents were inoculated three times in Vero cells and gently agitated at room temperature for 2 h. Supernatants were discarded and the MEM containing 0.3 % agarose (Invitrogen, USA) was overlaid. The cells were incubated at 37 °C, 5 % CO_2_ and CPE was daily observed until appearance of single plaques. Plaque number was counted after fixation of the cells with 4 % formaldehyde and staining with 0.2 % crystal violet to visualized single plaques clearly. The plaque number per millilitre was calculated.

### Virus infection

Vero and 293T cells were grown until the cells reached about 100 % confluence (approximately 1.5 × 10^6^ and 5.6 × 10^6^ cells/ml). The V-181 isolate and the isolated CHIKV were inoculated at a multiplicity of infection (MOI) of 10 in either the Vero or the 293T cells at room temperature with gently agitation for 2 h. Supernatants were removed and either infected Vero cell medium (MEM supplemented with 2 % heat-inactivated FBS) or infected 293T cell medium (DMEM supplemented with 2 % heat-inactivated FBS, 25 mM HEPES, 2 mM L-glutamine, and 0.37 % NaH_2_CO_3_) was added. The infected cells were incubated at 37 °C, 5 % CO_2_ for further experiments.

### Cytopathic effects

The cytopathic effects (CPE) of virus infection were studied using published procedures [23]. Vero cells infected with isolated CHIKV as described above were observed and scored three times for CPE under light microscopy with 5 different fields for 7 days on a daily basis. A CHIKV that induced the largest plaque size was selected for this study.

293T cells infected with either V-181 or the selected CHIKV isolate (CHIK/SBY8/10 herein abbreviated as T-SBY) were observed and scored three times for cytopathic effects (CPE) under light microscopy in 5 different fields for 10 days on a daily basis.

### Polymerase chain reaction and sequencing

To confirm the identity of the isolated clone of CHIKV, viral RNA from infected Vero cells from each clone were extracted using High Pure Viral RNA Kit (Roche, USA). RNA was used as a template for reverse-transcriptase polymerase chain reaction (RT-PCR) using the RevertAid First Strand cDNA Synthesis Kit (Thermo Fisher Scientific, USA). Viral non-structural protein 1 gene (*nsp1*) was amplified using the cDNA as a template. The primers used for PCR reactions are indicated in Table 1. To identify the selected CHIKV isolate, the 1317 base pair CHIKV *E1* envelope glycoprotein gene (*E1*) was amplified using the primers in Table 1. An amplicon was cloned into pJET 1.2/blunt cloning vector (Thermo Fisher Scientific, USA) and sequenced using an ABI 3730XL DNA Analyzer (Thermo Fisher Scientific, USA).

**Table 1:** List of primers used in this study for PCR and qRT-PCR

Primer	Forward primer	Reverse primer
CHIKV *nsp1*	5′-ccgctcgagtagagcaggaaattgatccc-3′	5′-cccaagcttctttaatcgcctggtggtat-3′
CHIKV *E1*	5′-ccgCTCGAGatgtacgaacacgtaacagtg-3′	5′-cccAAGCTTgtgcctgctaaacgacacgc-3′
Human *IL-6*	5′-gtagtgaggaacaagccagag-3′	5′-ggactgcaggaactccttaaa-3′
Human *COX-2*	5′-gacagtccaccaacttacaat-3′	5′-catctctccatcaattatctgat-3′
Human *MBL*	5′-ccgctcgagatgtccctg-3′	5′-cccaagcttgatagg-3′
Human *IL-1β*	5′-catgggataacgaggcttatgt-3′	5′-cccaaggccacaggtattt-3′
Human *IL-8*	5′-actgagagtgattgagagtggac-3′	5′- aaccctctgcacccagttttc-3′
Human *PLA-2*	5′-ctacctacgttgctggtctttc-3′	5′-ccaaataagtcgggagccataa-3′
Human *TNF-α*	5′-atcctgggggacccaatgtag-3′	5′-gagcttcttcccacccacaag-3′
Human *GAPDH*	5′-accacagtccatgccatcac-3′	5′-tccaccaccctgttgctgt-3′

### Sequence analysis

The *E1* nucleotide sequence of the selected CHIKV isolate was analyzed using NCBI BLASTX. Comparison of E1 amino acid sequence from the selected CHIKV isolate, V-181, and the parental V-181 strain (AF15561) was performed using ClustalW2 software [24].

### Virus infection studies in 293T cells

293T cells were infected with either CHIKV T-SBY or the V-181 strain at a MOI of 10 as described above. Unbound virus was removed and the cells were extensively washed thrice with DMEM supplemented with 2 % heat-inactivated FBS, 25 mM HEPES, 2 mM L-glutamine, and 0.37 % NaH_2_CO_3_. Freshly medium was added to the infected 293T cells. Medium was collected to measure viral load and RNA copy number at early (0, 2, 4, 6, and 12 h) and late (1, 2, 3, 4, 7, and 10 days) infection using a standard plaque assay and quantitative real-time PCR (qRT-PCR). Standard plaque assay and qRT-PCR were performed in three independent experiments.

### Indirect immunofluorescence assay

293T cells were infected with either CHIKV T-SBY or the V-181 strain at a MOI of 10 as previously described. Three independent experiments were performed. The infected cells were harvested at 0, 4, 7 and 10 d.p.i. by removing the infected 293T cell medium and gently washed the infected cells with Phosphate Buffer Saline (PBS) twice. The cells were detached using 0.25 % Trypsin-EDTA and washed with PBS at 1,500xg, at room temperature for 3 min thrice. Cell pellets were resuspended in PBS and smeared on a coverslip before air-drying at room temperature for 10 min. The cells were fixed with 4 % paraformaldehyde in PBS for 20 min at room temperature and washed with PBS. The cells were permeabilized with 0.1 % Triton X-100 in PBS for 15 min at room temperature and washed with PBS thrice. The cells were then incubated with 5 % normal goat serum in PBS with 0.05 % Tween 20 for 1 h at room temperature. Subsequently, the cells were incubated with a mouse anti-CHIKV monoclonal antibody (Thermo Fisher Scientific, USA) at a dilution of 1:10 at room temperature for 1 h. After washing with PBS thrice, the cells were incubated with a 1:100 dilution of a fluorescein isothiocyanate (FITC)-conjugated goat anti-mouse IgG polyclonal antibody (Thermo Fisher Scientific, USA). The cells were washed with PBS thrice and incubated with 500nM propidium iodide (PI) (Invitrogen, Ltd.) in PBS in the dark for 30 min at room temperature. After a brief wash with PBS, the cells were mounted with a mounting medium, covered with coverslips and sealed with nail polish. CHIKV infected 293T cells were observed under an Olympus DP-72 fluorescence microscope.

To determine the level of cell infection, CHIKV infected 293T cells were counted from five different microscopy fields in three independent experiments using ImageJ 1.49v software (National Institutes of Health, USA).

### Cell viability assay

Cell viability was measured using a classical MTT assay [25]. 293T cells were infected with either CHIKV T-SBY or the V-181 strain at MOI of 10 as previously described. Three independent experiments were performed. Infected cells were assessed for cell viability for 10 days on a daily basis. The infected 293T cell medium was removed and replaced with fresh medium. MTT (3-(4,5-Dimethylthiazol-2-yl)-2,5-Diphenyltetrazolium Bromide) was added to a final concentration of 1.3 mM and incubated at 37 °C for 4 h in a humidified chamber. The medium was removed and crystals were solubilized by adding dimethyl sulfoxide (DMSO) and incubating at 37 °C for 10 min. The samples were measured for absorbance using an ELISA reader at a wavelength of 570 nm.

### Quantitative real-time polymerase chain reaction

Quantitative real-time reverse transcription PCR (qRT-PCR) was used to determine viral RNA copy number and relative levels of cytokine gene expression. Supernatants from early (0, 2, 4, 6, and 12 h) and late (1, 2, 3, 4, 7, and 10 days) infections were prepared as described before. Viral RNAs were extracted by High Pure Viral RNA Kit (Roche, USA) and converted to cDNA using the RevertAid First Strand cDNA Synthesis Kit (Thermo Fisher Scientific, USA). The viral non-structural protein 1 gene (*nsp1*) was amplified using primers indicated in Table 1. Quantitative PCR analysis was performed using Luminaris Color HiGreen qPCR Master Mix, low ROX (Thermo Fisher Scientific, USA) as previously described [26]. The viral RNA copy number per ml was calculated. All quantitative PCR results were checked for specificity by melting curve analysis.

For cytokine gene expression, 293T cells were infected with either CHIKV T-SBY or the V-181 strain at a MOI of 10. Infected cells were harvested at 0, 4, and 7 d.p.i. and RNA was extracted using TRIzol reagent (Invitrogen, USA). RNA was used as a template for reverse-transcriptase polymerase chain reaction (RT-PCR) using the RevertAid First Strand cDNA Synthesis Kit (Thermo Fisher Scientific, USA). The primers used for PCR reactions are indicated in Table 1. The qRT-PCR was performed by Luminaris Color HiGreen qPCR Master Mix, low ROX (Thermo Fisher Scientific, USA). The target transcripts were normalized to the number of *GAPDH* transcripts for quantitative analysis of gene expression. Melting curve analysis was performed to checking for specificity.

### Statistical analysis

Results are expressed as the mean ± standard error of the mean (SEM). The significance of the difference between the mean values of the groups was evaluated by two-tailed Student’s *t* test.

## Results

### Isolation of a chikungunya virus isolate from human serum

**Fig. 1:**
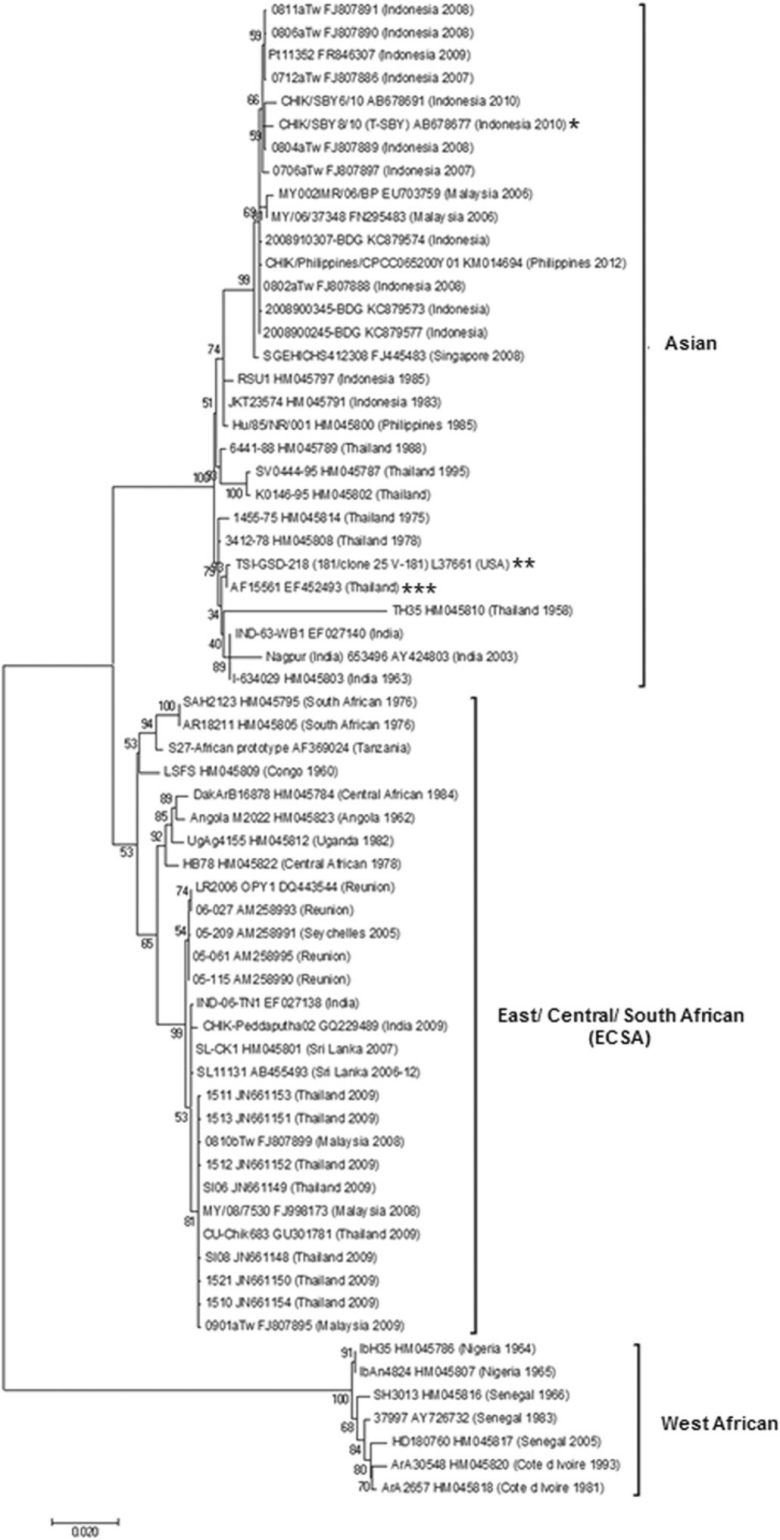
Molecular phylogenetic analysis by Maximum Likelihood method. The evolutionary history was inferred by using the Maximum Likelihood method based on the Tamura-Nei model [38]. The tree with the highest log likelihood (-4636.3022) is shown. The percentage of trees in which the associated taxa clustered together is shown next to the branches. Initial tree(s) for the heuristic search were obtained automatically by applying Neighbor-Join and BioNJ algorithms to a matrix of pairwise distances estimated using the Maximum Composite Likelihood (MCL) approach, and then selecting the topology with superior log likelihood value. The analysis involved 65 nucleotide sequences. All positions containing gaps and missing data were eliminated. There were a total of 1,317 positions in the final dataset. Evolutionary analyses were conducted in MEGA7 [39]. Asterisk (*) indicates the selected isolate from this study. Double asterisk (**) indicates the comparable strain in this study. Triple asterisk (***) indicates the parental strain of the comparable strain

To contribute to the biology and virulence of infectious CHIKV, we sought to isolate an infectious strain from human patients. To accomplish this, we collected banked serum samples from five patients from the 2009 outbreak in the Songkhla Province, Thailand, who were with tested negative for the presence of dengue virus (DENV) and yellow fever virus, as assessed by routine PCR analysis. CHIKV was isolated from each patient serum using standard procedures as detailed in the Materials and Methods section. The patients sera were inoculated in *Aedes albopictus* C6/36 cells and single clones were obtained by serial dilution before further propagation in Vero cells. After infection of the cells with five different isolates at a multiplicity of infection (MOI) of 10, plaque morphology and cytopathic effect (CPE) for each isolate was compared. The viral clone with highest plaque size and that induced most CPE virulence was selected for further study. To identify the strain of the selected CHIKV isolate, we synthesized cDNA from the viral RNA and amplified by PCR the 1317 base pair CHIKV *E1* envelope glycoprotein gene, which is usually used for identification of CHIKV strains and their phylogenetic analysis. Comparison of the *E1* nucleotide sequence using NCBI BLASTX revealed a close match with the E1 protein of the Indonesian CHIK/SBY8/10 isolate (GenBank accession number AB678677) with 100 % identity at the amino acid level and an E value of 0. Phylogenetic analysis of the E1 nucleotide sequence and the E1 sequences available in GenBank confirmed that the selected isolate belonged to the Asian genotype. It clustered within a clade of Indonesian sequences collected between 2008 and 2010 (Fig. 1). Hereinafter, we refer to this selected Thailand CHIKV isolate as T-SBY.

### Assessment of growth and release of T-SBY from 293T cells

**Fig. 2:**
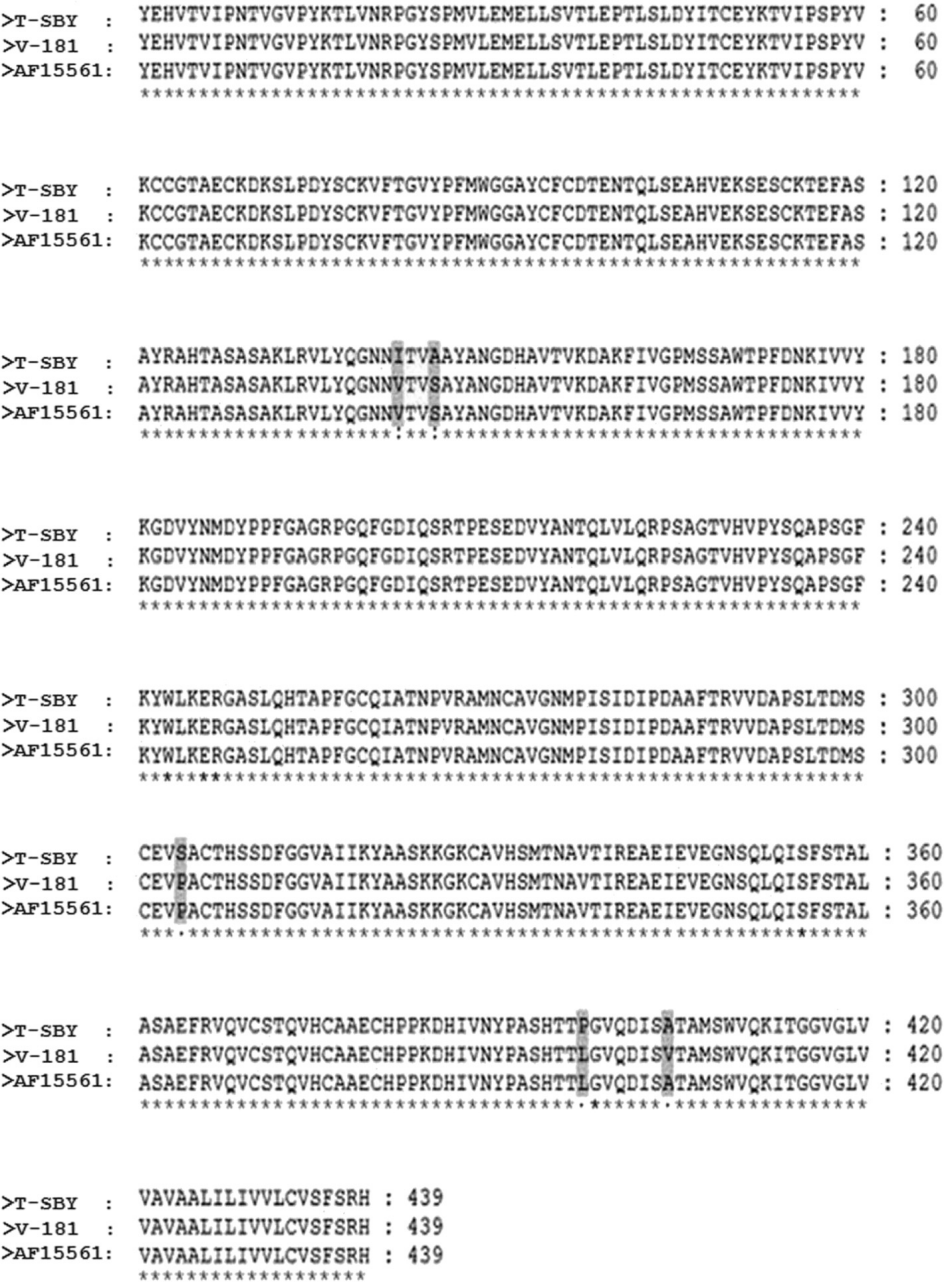
Multiple sequence alignment of CHIKV E1 envelope glycoprotein (E1). The 1,317 base pair CHIKV *E1* envelope glycoprotein gene from the isolated CHIKV was amplified by PCR. Comparison of the *E1* nucleotide sequence using NCBI BLASTX revealed a close match with the E1 protein in the CHIK/SBY8/10 isolate from Indonesia (GenBank accession number AB678677; T-SBY). Amino acid sequences of the E1 from T-SBY were aligned with E1 from the 181/clone 25 vaccine strain (GenBank accession number L37661; V-181) and its parental strain AF15561 (GenBank accession number EF452493) [27] using ClastalW2 software [24]. Asterisks (*) indicate completely identical amino acids. An alteration of amino acid residues is shaded in grey. Single (.) and double dot (:) represent semi-conservative and conservative amino acid substitution

We next studied the growth and release of the isolated virus in comparison with a well-studied CHIKV vaccine strain, called 181/clone 25, herein abbreviated as V-181. Of note, the vaccine strain V-181 (GenBank accession number L37661) was derived from the CHIKV parental strain AF15561 (GenBank accession number EF452493) as described in [27]. Analysis of the E1 envelope glycoprotein from the currently isolated strain, T-SBY, the vaccine strain V-181, and its parental strain AF15561 using ClastalW2 software [24] revealed two conservative amino acid substitutions at positions 142 and 145, from isoleucine (ATC) to valine (GTT), and alanine (GCT) to serine (TCT), respectively. Additionally, substitutions of three semi-conservative amino acids at positions, 304, 397, and 404 for serine (TCA) to proline (CCA), proline (CCC) to leucine (CTC), and alanine (GCT) to valine (GTT) were observed (Fig. 2). Note that the amino acid sequence of V-181 E1 envelope glycoprotein differs in a single amino acid substitution at position 404, from valine to alanine from the parental strain, AF15561 [27].

**Fig. 3:**
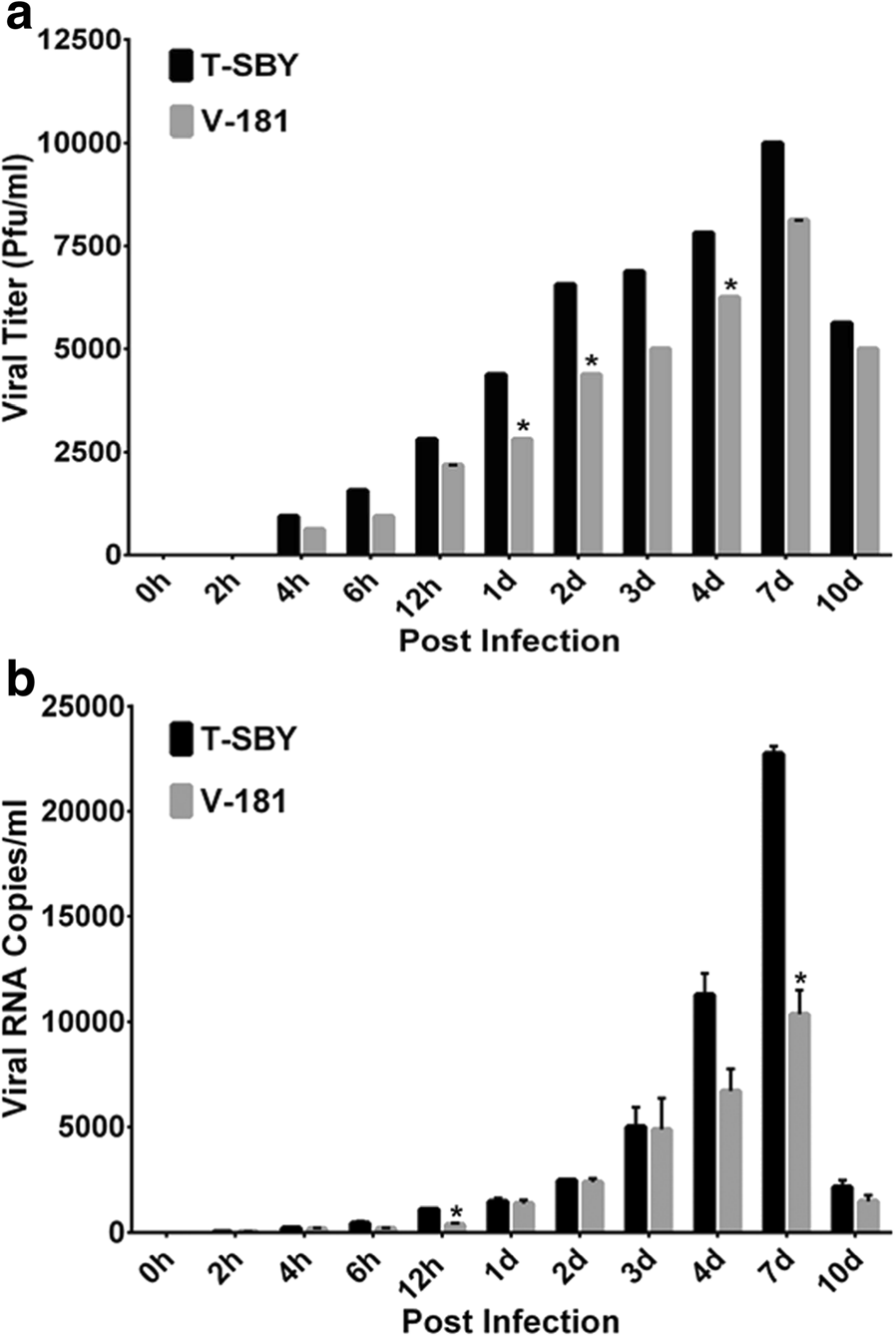
CHIKV release from 293T cells. 293T cells were exposed to either CHIKV T-SBY or V-181 for 2 h at a MOI of 10. Unbound virus was removed and washed extensively. Supernatants were collected for determination of viral loads (**a**) and viral RNA (**b**) at early (0, 2, 4, 6, and 12 h) and late (1, 2, 3, 4, 7, 10 d) infection using standard plaque assay and real time PCR as described in Materials and Methods. Results are expressed as viral titer (PFU/ml) (**a**) and viral RNA copies/ml (**b**). The bars represent the mean values and the error bars represent the SEM values from three independent experiments. Asterisks (*) indicate *p* < 0.05

As CHIKV can infect several types of cell lines [26, 28], we selected one of the permissive cells for chikungunya virus infection that support virus growth better when compare with other cell types, 293T cells. Notably, the 293T cells also are easy culture and maintain in laboratory conditions and are considered as a model cell line for various studies. We therefore, selected the 293T cells, as a model in this study. To examine the growth and release of the isolated virus in comparison with CHIKV V-181, we determined the viral load and RNA copy number at different time points following infection of 293T cells (Fig. 3). The cells were exposed to either CHIKV T-SBY or V-181 strain for 2 h at a MOI of 10. The culture was washed extensively to remove any unbound virus. The viral load was determined in the supernatant from three independent experiments at early time points (0, 2, 4, 6, and 12) hours post infection (h.p.i.) as well as during late time points (1, 2, 3, 4, 7, and 10) days post infection (d.p.i.) using standard plaque assay as described in Materials and Methods. The results showed that in the first 2 h.p.i. there was no detection of infectious virus from cells infected with either strain, further testifying complete removal of virus in the supernatant. ﻿However,﻿ the levels of newly produced progeny viruses were gradually increased in the supernatants after 4 h.p.i, reaching  the maximum levels of ~10^4^ and ~8 × 10^3^ PFU/ml in CHIKV T-SBY and V-181 strains, respectively, after 7 d.p.i. No statistically significant was observed. Thereafter, the viral levels decreased at 10 d.p.i, and no virus in supernatants could be detected at 15 and 20 d.p.i. (data not shown). At all post infection periods, the T-SBY levels were higher than the V-181 although statistically significant differences were recorded at 1, 2, and 4 d.p.i. (Fig. 3a).

In a parallel set of experiments, we also quantified viral RNA in the supernatant from three independent experiments at early post infection (0–12 h.p.i.) and late post infection (1–10 d.p.i.) using an established real-time PCR assay [26]. We did not normalize the virus RNA copies with cell GAPDH copy number as such normalization would be biased by the state of cellular transcription or GAPDH levels in the cell. There are likely to be a substantial fraction of infected cells with a high virus titer but with limited transcription or degraded GAPDH levels. The result was overall consistent with virus release in supernatant with only minor differences (Fig. 3b). The viral RNA copies could be detected in both infectious T-SBY and V-181 supernatants at 2 h.p.i. with 28 ± 1 and 35 ± 6 viral RNA copies/ml in infectious T-SBY and V-181 supernatants, respectively. The amount of viral RNA copies gradually increased and reached the peak level of greater than 10^4^ RNA copies/ml in both T-SBY and V-181 infected supernatants at 7 d.p.i, and then declined at 10 d.p.i. The number of T-SBY viral RNA copies was consistently higher than that of V-181 at almost all post infection time points with statistically significant differences at 12 h.p.i. and 7 d.p.i. (Fig. 3b). Similar to the plaque assay, no viral RNA in supernatants could be detected at 15 and 20 d.p.i. (data not shown).

### Cytopathic effect of T-SBY in 293T cells

**Fig. 4:**
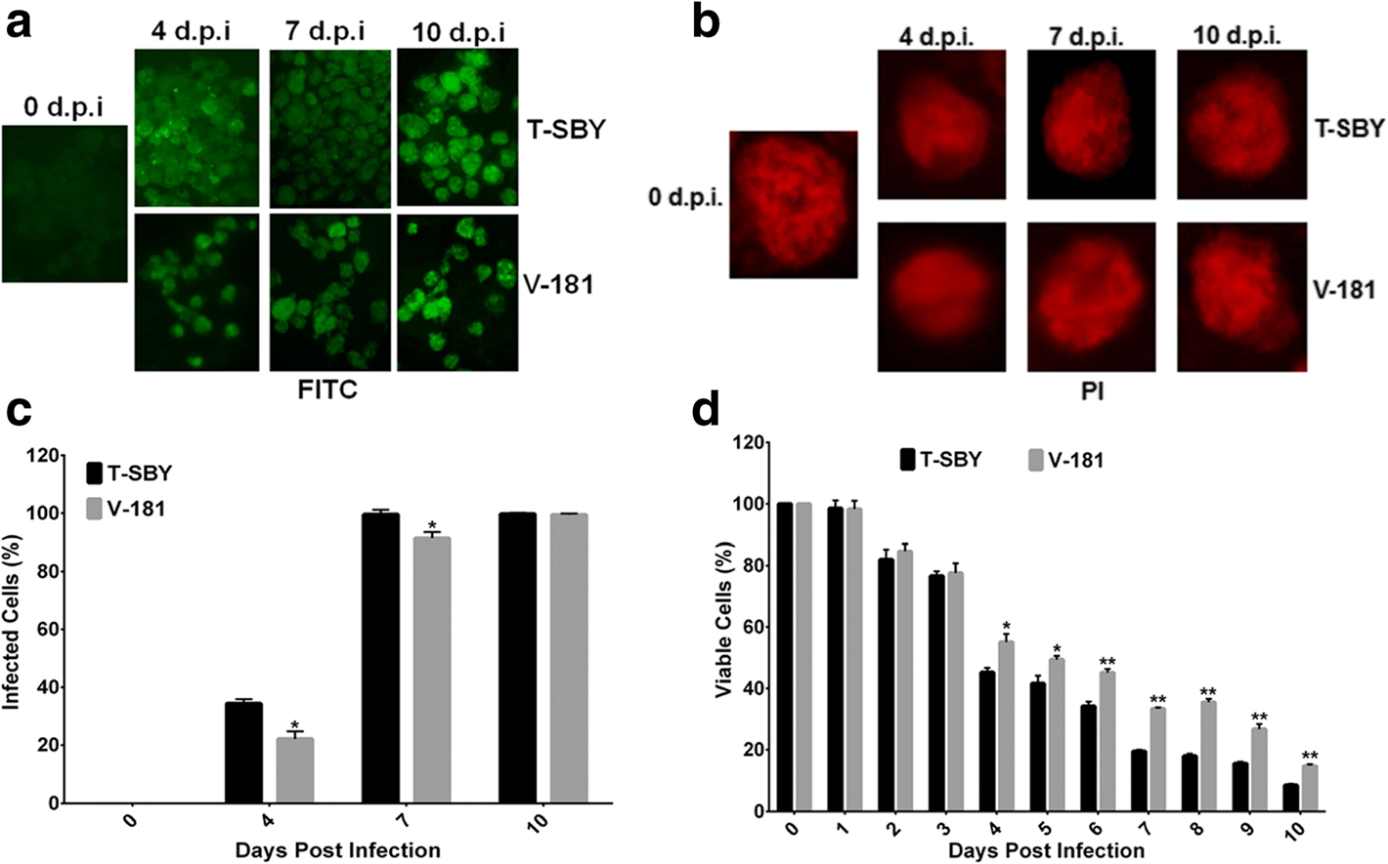
CHIKV infection of 293T cells. (**a**) 293T cells were infected with either CHIKV T-SBY or V-181 at MOI of 10. Infected cells at 0, 4, 7, and 10 d.p.i. were collected and stained with antibody against CHIKV and FITC-labeled secondary antibodies to visualize the infected cells at 0, 4, 7, and 10 d.p.i. (**b**) 293T cells were infected with either CHIKV T-SBY or V-181 as described in panel A. Infected cells were collected and stained with propidium iodide (PI) to visualize the nuclei prior examination under a fluorescence microscope. Foci were observed at 0, 4, 7, and 10 d.p.i. (**c**) 293T cells were infected as detailed in panel A. Infected cells at 0, 4, 7, and 10 d.p.i. were collected and labeled with an anti-CHIKV monoclonal antibody followed by an appropriated FITC-labeled secondary antibody as well as propidium iodide (PI) to visualize the nuclei prior examination under a fluorescence microscope. Quantification of CHIKV infected cells were analyzed using ImageJ 1.49v software after immunostaining. Three independent experiments were performed and the bars represent the mean values and the error bars represent the SEM values. The amount of CHIKV T-SBY infected cells was statistically significantly higher (*p < 0.05) at 4 and 7 d.p.i. (**d**) Analysis of cell viability using a classical MTT assay. The 293T cells were exposed with either CHIKV T-SBY or V-181 for 2 h at an MOI of 10. Infected cells were assessed for cell viability during 10 days on a daily basis. The bars represent the mean values and the error bars represent the SEM values from three independent experiments. The difference in cell viability in CHIKV T-SBY infected cells was statistically significant (*p < 0.05) at 4 and 5 d.p.i., whilst it was highly significant (**p < 0.001) at 6 d.p.i.

To compare the development of infection of CHIKV T-SBY with that of V-181, 293T cells were infected with either strain at a MOI of 10. Cytopathic effects (CPE) were observed under light microscopy daily for 10 days (data not shown) when cells became most infected, as revealed by the highest immunoreactivity (Fig. 4a). By 3 d.p.i., about 10 ± 0.7 and 3 ± 0.5 % of total infected T-SBY and V-181 cell number became swollen, respectively. The increase of cell diameter continued within the next day, accounting for an additional increase of 25 ± 1.5 and 10 ± 2 % at 4 d.p.i, respectively. In addition, the infected cells detached from the surface of the culture plate with formation of small black spot in the cells (Fig. 4b). Notably, at all time points these spots were more pronounced in cells infected with T-SBY than in those infected with V-181. The amount of swelled and detached cells and black spot formation continued to increase until they reached 80 ± 5.0 and 60 ± 2.5 % in cultures infected with T-SBY and V-181, respectively, by 10 d.p.i. Therefore, in summary, the cells infected with T-SBY were more swelled and detached more quickly, compared to cells infected with V-181.

To visualize the development of infection, 293T cells were infected with either CHIKV T-SBY or V-181 at a MOI of 10 and processed for an immunofluorescence assay with a mouse anti-CHIKV monoclonal antibody at 0, 4, 7, and 10 d.p.i. At 4 d.p.i., about 34 ± 2 and 22 ± 3 % of the cells infected with CHIKV T-SBY and V-181 strains, respectively, showed expression of the viral antigen (Fig. 4c). By 7 d.p.i., infection could be detected in approximately ~100 and ~91 % of cells infected with CHIKV T-SBY and V-181 strains, respectively. No statistically significant difference was observed.

We also determined cell viability with a classical MTT assay as described in Materials and Methods. The cells were infected with either CHIKV T-SBY or V-181 at a MOI of 10. The viability of the infected cells was determined daily for 10 days. The viability of cells infected with CHIKV T-SBY was lower than that of cells infected with V-181 cells starting at 2 d.p.i. with statistically significant (*p < 0.05) at 4 and 5 d.p.i., and highly statistically significant differences (**p < 0.001) from 6 to 10 d.p.i. (Fig. 4d).

### Expression of cytokines during T-SBY infection of 293T cells

**Fig. 5:**
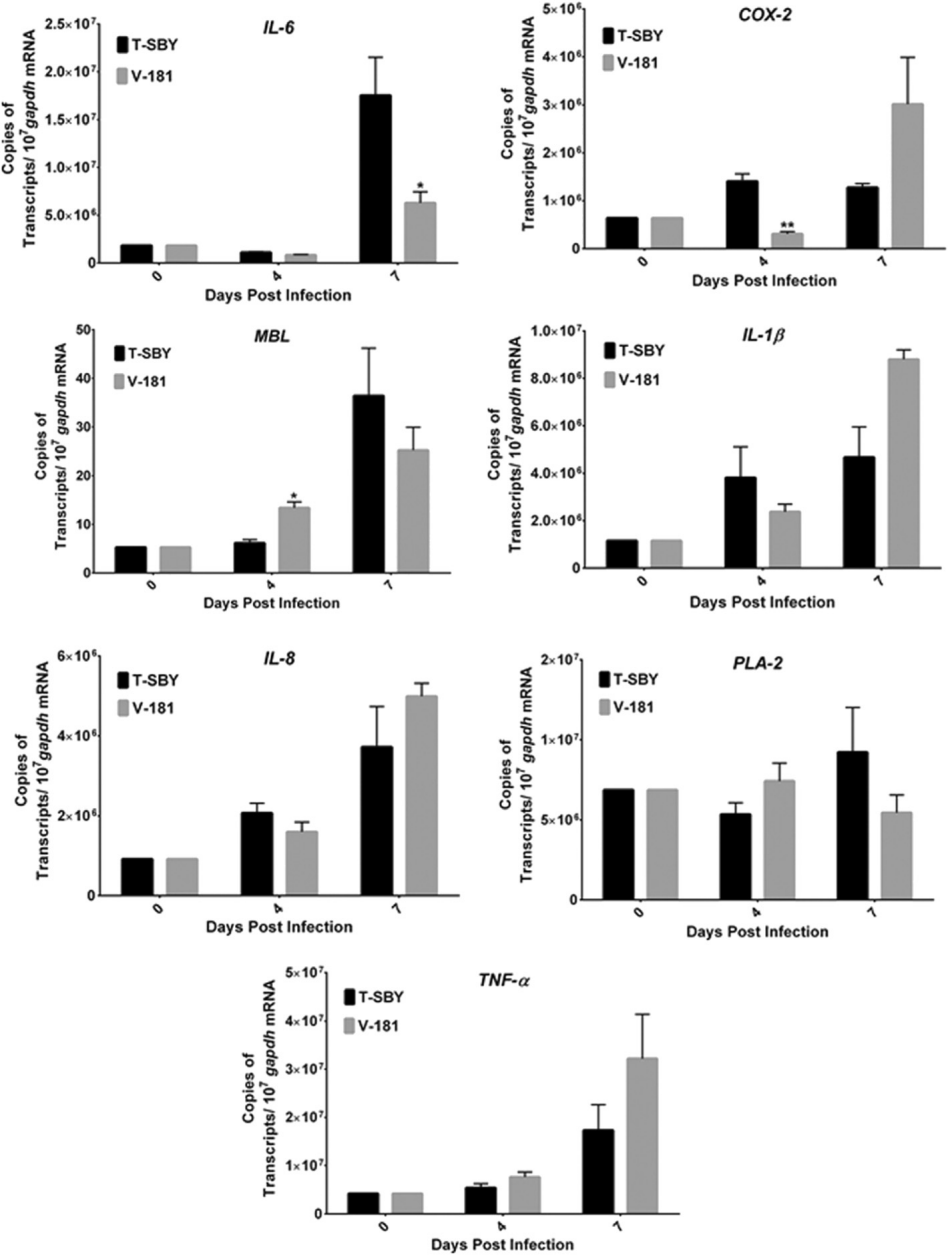
Expression of cytokine gene candidates during CHIKV infection of 293T cells. The relative expression levels of *IL-6, COX-2, MBL, IL1-β, IL-8, PLA-2*, and *TNF-a* during CHIKV infection of 293T cells were analyzed. Transcript levels of candidate genes were measured using quantitative RT-PCR and presented as copies of target transcripts per copy of the *GAPDH* transcripts. RNA was isolated from CHIKV infected cells at 0, 4, and 7 d.p.i. The bars represent the mean values and the error bars represent the SEM values from six quantitative RT-PCR analyses of two independent cell infection experiments. The relative expression levels of *IL-6* and *MBL* in CHIKV T-SBY infected cells were statistically significantly higher (*p < 0.05) at 7 and 4 d.p.i., respectively, whilst the difference in the relative expression levels of *COX-2* was highly significant (**p < 0.001) at 4 d.p.i.

As previous results suggest that the our T-SBY strain isolated from a patient is more infectious and induces stronger cytopathic effects upon infection of 293T cell than the vaccine strain V-181, we were interested to examine virus-induced inflammatory cellular responses [26, 29]. CHIKV is known to induce cellular inflammatory responses and the level of certain cytokines in CHIKV-infected patient serum has been shown to correlate with the symptomatic phase of infection. We therefore wanted to study the expression of selected cytokine genes that were previously shown to be activated during symptomatic phase of CHIKV infection and involved in the genesis of inflammatory host responses, including rheumatoid arthritis (RA) [30]. 293T cells were infected with either CHIKV T-SBY or V-181 at a MOI of 10. Total RNA was isolated from infected cells at 0, 4, and 7 d.p.i. Transcription levels of Interleukin-6 (*IL-6*), Cyclooxygenase-2 (*COX-2*), Mannose binding lectin (*MBL*), Interleukin1-beta (*IL1-β*), Interleukin-8 (*IL-8*), Phospholipase A2 (*PLA-2*), and Tumour necrosis factor alpha (*TNF-α*) were measured using quantitative RT-PCR and normalized per *GAPDH* transcripts. Results showed that both viruses induced expression of all examined cytokine or inflammation-related genes. Although levels of induction of cellular inflammatory responses were similar for both viruses, statistically significant higher expression of *COX-2*, *MBL,* and *IL-6* was observed in cells infected with T-SBY at 4 and 7 d.p.i. (Fig. 5). Although the precise mechanisms of how these two CHIKV strains induce cellular inflammatory responses or biological significance of their noticeably different cytokine response profiles awaits future investigation, our study further underscores the utility of 293T cells for studying the biology and cellular responses to CHIKV infection.

## Discussion

Chikungunya virus (CHIKV) is one of the recently emerging and re-emerging serious mosquito-borne pathogen that can elicit significant human morbidity in many parts of the world [1]. The virus is responsible for recent large-scale epidemics in many tropical and sub-tropical countries, including one in the southern provinces of Thailand, where 49,069 human cases were reported in little over a year between 2008–2009 [20]. Notably, subsequent studies identified variations among the Thai isolates, including clones harbouring unique amino acid substitutions close to the immunodominant site [21]. Compared to many other arthropod-borne infective agents, CHIKV is relatively less well studied, which is now considered as an important neglected tropical disease [31]. While recently significant efforts were directed towards a better understanding of the biology, infectivity and pathogenesis of CHIKV infection, as a new health global risk, including 693,489 suspected cases reported alone in the Americas in 2015 [32], further studies on the biology and virulence of CHIKV remain warranted, especially those focused on the virus isolated from patients from recent epidemics. In this report, we isolated a CHIKV strain from patients’ sera banked from the 2008–2009 Thai epidemics. We identified this virus is closely related to the Asian genotype, CHIK/SBY8/10 isolate originally found in patient’s blood in Indonesia [33]. To the best of our knowledge, no prior study addressed the characterization, replication, and release of the strain used in our study. Therefore, our study likely represents the first characterization of this clinical strain. We used 293T cells as in vitro model for studying viral growth kinetics, inflammatory cellular responses, and virus-induced cytopathic effects. Based on the release of viral particles in the supernatants or measurement of viral mRNA from supernatants, viral replication could be detectable within 4 h of infection, and continued to increase gradually peaking at 7 days. A massive detachment of CHIKV-infected cells after 10 days post-infection suggested virus-induced cell death, possibly via apoptosis as reported in Vero cells [26, 28]. The decline of virus titer or RNA copies at 10 d.p.i. clearly demonstrated a decrease of viral replication, which may be due to the lysis or death of infected host cells. Similarly, due to absence of viable cells supporting growth of CHIKV, undetectable virus titer or RNA copies at 15 and 20 d.p.i. likely indicated disappearance of the virus.

Based on the growth kinetics, microscopic observation, CPE, and cell viability T-SBY is more virulent than the vaccine strain V-181 used in our study. A single amino acid alteration in the E1 surface glycoprotein was shown to have profound effects on CHIKV host tropism [27], we found 4 amino acid changes in the E1 protein between T-SBY and the vaccine strain V-181 which can explain why T-SBY is more virulent, however the precise role of any of these substitutions as well as the potential input of the differences in other viral proteins awaits further investigation.

The arthritogenic potential of CHIKV is associated with its ability to persist in the joint tissue and induce the production of proinflammatory cytokines. Indeed, CHIKV infection induces a potent inflammatory cytokine and immunomodulatory response in patients, with more severe disease associated with increased plasma levels of several proinflammatory cytokines [11], particularly IL-6 [34]. Many of these cytokines are associated with the pathogenesis of systemic inflammatory disease, such as rheumatoid arthritis [35, 36], which is one of the most serious clinical complications of CHIKV fever. We therefore, also examined the induction of several cytokines and immunomodulators in 293T cells. The majority of these cytokines and immunomodulators displayed a gradually enhanced transcription during the course of 293T cell infection, which correlated with increased viral loads. While the induction of these proinflammatory modulators by the two different CHIKV isolates was very similar, the expression of *IL-6* and *COX-2*, was statistically significantly higher during infection with the more virulent isolate, T-SBY, especially late during infection (between day 4–7). While induction of *COX-2* is known to be transient and its pro-inflammatory role remains controversial [37], our study suggests a possible contribution of this immunomodulator to enhanced virulence of at least some CHIKV isolates.

## Conclusions

In summary, we describe the successful isolation of a CHIKV strain, which was identified based on the *E1* envelop glycoprotein sequence. The isolated virus is closely related to the CHIKV isolate SBY8/10 identified in patients in Indonesia. We determined that 293T cells are an excellent cell culture model of CHIKV SBY8/10 that supports viral replication, development of CPE and subsequent cell death. We also determined expression of proinflammatory cytokines and immunomodulatory molecules upon CHIKV infection of 293T cells, which can be involved in CHIKV-induced immune responses and contribute to the immunopathology of the viral infection.

## Abbreviations

AF15561: The parental V-181 strain
AFRIMS: The Armed Forces Research Institute of Medical Sciences
ATCC: American Type Culture Collection
CHIKV: Chikungunya virus
*COX-*2: Cyclooxygenase-2
CPE: Cytopathic effects
d.p.i.: Days post infection
DMEM: Dulbecco’s Modified Eagle Medium
DMSO: Dimethyl sulfoxide
*E1*: *E1* envelope glycoprotein gene
FBS: Fetal bovine serum
FITC: Fluorescein isothiocyanate
h.p.i.: Hours post infection
*IL1-β*: Interleukin1-beta
*IL-6*: Interleukin-6
*IL-8*: Interleukin-8
*MBL*: Mannose binding lectin
MEM: Minimum Essential Medium
MOI: Multiplicity of infection
MTT: 3-(4,5-Dimethylthiazol-2-yl)-2,5-Diphenyltetrazolium Bromide
nsp: Nonstructural protein
*nsp1*: Non-structural protein 1 gene
PBS: Phosphate Buffer Saline
PCR: Polymerase chain reaction
PI: Propidium iodide
*PLA-2*: Phospholipase A2
qRT-PCR: Quantitative real-time PCR
RA: Rheumatoid arthritis
RT-PCR: Reverse-transcriptase polymerase chain reaction
SEM: Standard error of the mean
*TNF-α*: Tumour necrosis factor alpha
T-SBY: CHIK/SBY8/10
V-181: CHIKV 181/clone 25
